# Tell Me a Story: Socio-Emotional Functioning, Well-Being and Problematic Smartphone Use in Adolescents With Specific Learning Disabilities

**DOI:** 10.3389/fpsyg.2019.02369

**Published:** 2019-11-06

**Authors:** Daniela Sarti, Roberta Bettoni, Ilaria Offredi, Marta Tironi, Elisabetta Lombardi, Daniela Traficante, Maria Luisa Lorusso

**Affiliations:** ^1^Fondazione IRCCS Istituto Neurologico Carlo Besta, Milan, Italy; ^2^Department of Psychology, University of Milano-Bicocca, Milan, Italy; ^3^Department of Psychology, Catholic University of the Sacred Heart, Milan, Italy; ^4^Istituto IRCCS Eugenio Medea, Lecco, Italy

**Keywords:** socio-emotional functioning, well-being, smartphone addiction, SLD, adolescence, narratives

## Abstract

Although Specific Learning Disabilities (SLD) are described as specific difficulties in one or more academic areas, often socio-emotional problems are also reported to be related to well-being and school engagement. Moreover, recent evidence shows that emotional problems and reduced social support predict problematic use of new technologies, such as a smartphone, that can, in turn, increase these problems. In this study, we aimed to investigate socio-emotional functioning and its relation to well-being, school engagement, and problematic smartphone use. Social and emotional skills of 19 adolescents with a diagnosis of SLD and 19 control adolescents were assessed through a narrative test; adolescents were requested to narrate complete stories elicited by pictures representing social situations. Information on well-being and problematic smartphone use were collected through questionnaires. The comparison between groups showed differences in cognitive-social skills, although no significant differences in emotional functioning were found. Additionally, the perception of the social environment as supportive and trustworthy was related to general well-being for both groups, whereas the perception of limits and rules set by the adult world appeared to be related to a decreased investment in learning processes only for the SLD students. Finally, correlation analysis showed that smartphone use was associated with reduced perception of social support and to a decreased ability to understand and solve social situations. These results assert the critical role played by social information processing and social support in terms of well-being in adolescence, and contribute to enhancing knowledge of the mechanisms underlying problematic smartphone use in a clinical sample.

## Introduction

In the last 20 years, the use of technology is constantly increasing and has become very common, especially among adolescents (De Pasquale et al., 2017) who are progressively encouraged to use technological devices for learning purposes. Nevertheless, the use of technology needs to be adapted to the increasing heterogeneity of students, who have different cultural and linguistic backgrounds and different neuropsychological profiles (Lawless and Pellegrino, 2007; Perelmutter et al., 2017). In Italy, 2.5–3.5% of students have been diagnosed with Specific Learning Disabilities (SLD), such as dyslexia, dyscalculia, dysgraphia, and dysorthography (MIUR, 2015). SLD are neurodevelopmental disorders characterized by difficulties in specific academic areas despite average intelligence and adequate educational and socio-cultural opportunities (American Psychiatric Association [APA], 2013). In addition, it has been reported that students with SLD often exhibit social-emotional problems (i.e., Mugnaini et al., 2009; Francis et al., 2018), such as an increased risk of developing externalizing and internalizing problems, loneliness, and poor self−esteem (Bryan, 2005; Wilson et al., 2009; Aro et al., 2019). Social competence also seems to be a challenge for many SLD students, especially during adolescence when social functioning becomes increasingly complex and multifaceted (Cosden et al., 2002; Mugnaini et al., 2009). For example, it has been reported that SLD students experience low-quality relationships with their peers, higher rejection, and lower acceptance (Wiener and Schneider, 2002; Al-Yagon and Margalit, 2013), they are less likely to report a secure attachment with their parents, and they have difficulties in perceiving teachers as a secure base (Al-Yagon, 2012). Some researchers suggest that these difficulties in social competence may be a result of the social stigma frequently attached to SLD (Chan et al., 2017) and, in general, to other populations affected by neurodevelopmental disorders (i.e., intellectual disabilities; Ali et al., 2012). Specifically, due to their poor academic performance, students with SLD are often perceived as being less attractive, less successful, and lazier than their peers and, therefore, they may tend to believe and internalize this biased perspective about themselves. This can, in turn, affect the way they behave in social contexts (e.g., Livingston et al., 2018). Some researchers have also examined the mental processes that underlie the understanding of social interactions, investigating how social cues are encoded, interpreted, and how potential responses are generated (Crick and Dodge, 1994). Recent studies have focused on these aspects of children with and without SLD. Using social vignettes described by the examiner, participants are asked to answer a series of questions about different aspects of social information processes; that is, to recognize the problem in the vignette, to clarify goals, to make a response decision, and to evaluate given alternative solutions. SLD students have shown poorer ability to encode social cues, to produce alternative solutions, and to select the most feasible competent one (Bauminger and Kimhi-Kind, 2008; Bauminger-Zviely et al., 2019).

Social competence and perceived support are particularly important because they are related to academic success and engagement (Welsh et al., 2001; Estell and Perdue, 2013). Moreover, acceptance by peers is an essential part of adolescent self-identity and has a strong influence on psychological well-being (La Greca and Harrison, 2005). Difficulties in this area of functioning compounded by a struggle to achieve adequate school results could negatively influence school commitment and general well-being. Unfortunately, these aspects are still scarcely investigated with adolescents.

When discussing adolescents and peer relationships, it is necessary to consider the use of smartphones since they have become an essential part of student lives. Smartphone applications have been proven to be useful for youth in various aspects, including health-related smartphone applications (Do et al., 2018) and beauty and nutrition counseling (Tran et al., 2018). However, the majority of smartphone use is spent on social activities (e.g., using Instagram, Facebook, and Snapchat), especially among Italian adolescents (ISTAT, 2018). This seems to be grounded in a deep evolutionary need to be connected with others and suggests a prosocial nature of smartphone usage (Veissière and Stendel, 2018) which can, in turn, have a positive effect on subjective well-being (Teppers et al., 2014). However, this depends on the amount of time spent on mobile phones and social networks (Twenge, 2019). Increased use of this device has been connected with the perception of diminished quality relationships with classmates (Wang et al., 2017), lower satisfaction with life (Lepp et al., 2014) and poor sleep quality (Zhang et al., 2017). Recent studies have shown that emotional stress and low academic performance seem to mediate the relationship between excessive smartphone use and well-being (Samaha and Hawi, 2016; Hawi and Samaha, 2017). Lack of social support also seems to play an important role in predicting problematic smartphone behaviors, and this relationship has negative consequences on well-being (Herrero et al., 2019). Students that perceive a low level of social support seem to engage more often in smartphone activities that can satisfy their necessity of social connections. In addition, the abuse of smartphones negatively affects the perception of social support over time. A low level of social support together with smartphone addiction can lead to higher levels of psychological distress (Van Deursen et al., 2015; Kim, 2017; Wang et al., 2017; Herrero et al., 2019). In this context, it is possible to hypothesize that students with SLD represent a population vulnerable to the problematic use of a smartphone, as they have reported more internalizing and externalizing problems and more difficulties in reaching satisfying, supportive relationships (Francis et al., 2018; Bauminger-Zviely et al., 2019).

Research on this topic among the SLD Italian population is still scarce, especially about how social support, emotional distress, well-being, and addiction are related to each other. Using a performance-based test, we aimed to investigate whether SLD Italian students are less skillful in understanding and solving social situations, manifest more emotional problems, and perceive less social support than the control group. Moreover, considering the brief review of scientific literature aforementioned, we hypothesized that general well-being and school engagement of both SLD students and the control group could be related to emotional and socio-cognitive functioning. Finally, since extensive use of smartphones could be related to emotional and socio-cognitive functioning for both SLD and the control group (e.g., Herrero et al., 2019), we explored if SLD represents an at-risk population for smartphone addiction.

To empirically verify these hypotheses, high school students with and without a diagnosis of SLD were tested. Narrative test Roberts-2 (Roberts and Gruber, 2005; for Italian validation: Parolin et al., 2019) was used to provide an index of social cognitive understanding and emotional functioning, while general well-being and school engagement were assessed using self-report measures (“Comprehensive Inventory of Thriving-CIT,” Su et al., 2014; “Student Engagement Scale,” Mameli and Passini, 2017). Usually the perception and understanding of everyday interpersonal situations and their intrapersonal implications are assessed through self-report instruments or interviews, responses to which can be affected by misrepresentation and poor self-awareness (Hopwood and Bornstein, 2014). In addition to self-report measures, we used a performance-based test to better control for response bias and to maximize the imprint of individuality, since the students were asked to produce their own stories. Given the growing awareness of smartphone impact on social functioning and the heightened vulnerability to addiction during adolescence, we also included a measure of smartphone use (“Smartphone Addiction Scale,” Kwon et al., 2013; for Italian standardization: De Pasquale et al., 2017) in order to examine its link with socio-emotional functioning. Although the literature on this topic is still scarce, a greater understanding of adolescents’ socio-emotional functioning and its relation to well-being and smartphone use can be of paramount importance for adolescents with SLD because these aspects may operate as risk factors for them.

## Materials and Methods

### Participants

The sample is composed of 19 adolescents (male = 5) with a diagnosis of SLD group and with a mean age of 15.16 years (*SD* = 0.36) and 19 typically developing adolescents (CNT group; male = 9) with a mean age of 15.42 years (*SD* = 0.77). All participants were recruited from three high schools located near Milan that were part of the project “New technologies for education and their impact on students’ well-being and inclusion.” We included students from Scientific Lyceum (21%), Technical School (39.5%), and Vocational School (39.5%). Gender distribution was different for these curricula (χ^2^ = 19.16; *p* < 0.001), with girls preferring the Technical Schools (54%) and boys preferring Scientific Lyceum (57%).

All participants voluntarily took part in the study after parents gave their informed consent. The study was approved by the Ethics Committee of Catholic University of the Sacred Heart, according to standards of the Helsinki Declaration (World Medical Association, 2013). Adolescents included in the study met the following inclusion criteria: they were fluent Italian speakers and they obtained an intelligence score within the normal range on a standardized intelligence test (IQ > 25 centiles; Raven’s Colored Matrices test, Raven, 2000). All participants with SLD had received a clinical diagnosis based on standard inclusion and exclusion criteria (DSM-V; American Psychiatric Association [APA], 2013). Five students had received a diagnosis of dyslexia, five of dyscalculia, one of dysgraphia, and seven of mixed disabled scholastic skills, and for all of them, Individualized Education Programs were applied in the school context. Comorbidity with other psychopathological conditions were excluded. In order to be included in the CNT group, adolescents had to obtain a score within the normal range (>−1.5 SD) in reading speed, accuracy, passage reading comprehension, and dictation scores, which were obtained through the administration of the following Italian standardized tests: (1) Test of Word and non-word reading tests, drawn from “Batteria per la valutazione della Dislessia e della Disortografia Evolutiva-2, DDE-2” (Battery for the Evaluation of Dyslexia and Evolutionary Disorthography-2; Sartori and Job, 2007). Norms for high school students were based on a study by Arina et al. (2013). (2) Passage reading comprehension test and dictation test, drawn from Prove MT Avanzate-3-Clinica (MT 3 Advanced; Cornoldi et al., 2017). Between SLD and CNT groups, there were no group differences in age: SLD group, M = 15.42, SD = 0.77; CNT group, *M* = 15.16, *SD* = 0.36; *t*(36) = 1.34; *p* = 0.188.

Participant characteristics compared with a series of *t*-tests are shown in Table 1. Reflecting the recruitment criteria, the SLD group differed significantly from the CNT group in all reading and orthography scores.

**TABLE 1:** Participants characteristics that reflected the recruitment criteria.

**Test**	**CNT group (*n* = 19)**	**LD group (*n* = 19)**	***t*-test**	***P***	**Cohen’s *d***
Word reading test accuracy	0.36 (0.56)	−1.43 (2.26)	3.34	0.002	1.11
Word reading test speed	0.24 (0.56)	−1.03 (1.20)	4.18	>0.001	1.40
Non-word reading test accuracy	0.37 (0.76)	−0.78 (1.45)	3.07	0.004	1.02
Non-word reading test speed	0.18 (0.58)	−1.02 (0.92)	4.82	>0.001	1.61
Reading Comprehension test	0.56 (0.78)	0.03 (0.86)	1.99	0.054	0.66
Orthography test accuracy	0.56 (0.56)	−0.26 (1.11)	2.89	0.006	0.96

*The two groups differed significantly in all reading scores and in the orthography test.*

### Procedure

The study took place during school hours. For each participant, the session lasted about 2 hours. The battery comprised the administration in Italian of self-report and performance-based tests: two questionnaires were used to measure well-being both from a holistic point of view CIT(CIT; Su et al., 2014) and in the school context (Student Agentic Engagement Scale; Mameli and Passini, 2017); one questionnaire was used to account for problematic smartphone use (SAS-SV; De Pasquale et al., 2017); and assessments of general cognitive ability, language, and learning skills along with narrative tests were used to evaluate the ability to understand and resolve social problems (Roberts-2 test; Parolin et al., 2019). Participants engaged in questionnaires collectively, whereas cognitive, language, learning measures, and Roberts-2 tests were administered individually in a quiet and well-lit room provided by the school.

### Measures

#### Roberts-2 Test

Roberts-2 is a performance-based narrative test for children and adolescents 6 to 18 years old (Roberts and Gruber, 2005 for Italian version, see: Parolin et al., 2019). The test is composed of 27 black and white stimulus cards differentiated based on gender. The final set for each participant is composed of 16 pictures. The pictures depict social situations of everyday life both with peers and family. For each image, the participant is asked to create a complete story, composed by a beginning, a description of what is happening in the picture, an end, and a description of the characters’ feelings. The scoring system allows for the evaluation of the content of each story according to specific areas of functioning. The first area refers to the narrative components necessary to complete the task [scales: Complete Meaning (MEAN), Problem Identification (PID2, PID3, PID4), and Resolution (RES2, RES3, RES4, RES5)]. These variables refer to cognitive psychological functions that enable the adolescent to build a story with a complex and elaborated structure. The second area of functioning is evaluated by scales that measure emotional content and the resources on which the adolescent can rely to solve the problem depicted in the picture [scales: Support Self Feelings (SUPS-F), Support Self Advocacy (SUPS-A), Support Other Feelings (SUPO-F), Support Other Help (SUPO-H), Reliance on Other (REL), Limit Setting (LIM), Anxiety (ANX), Depression (DEP), Aggression (AGG), Rejection (REJ), and Unresolved Outcome (UNRS)]. These scales evaluate the ability to organize stories and include the possibilities to ask for help in a supportive environment, to rely on personal resources, and to take into consideration emotions and feelings.

#### Comprehensive Inventory of Thriving

The CIT is a validated self-report questionnaire composed of 54 items assessing seven dimensions of psychological well-being: Relationship, Engagement, Mastery, Autonomy, Meaning, Optimism, and Subjective Well-Being. A total score (Su et al., 2014) is also obtained. Participants were instructed to respond to each item on a scale of 1 (“Strongly Disagree”) to 5 (“Strongly Agree”).

#### Student Engagement Scale

The Student Engagement Scale is composed of 43 items measuring student engagement in school in four dimensions: affective, behavioral, cognitive, and agentic (Mameli and Passini, 2017). The items are rated on a 7-point Likert scale of frequency or agreement (from 1 = never/strongly disagree to 7 = always/strongly agree).

#### Smartphone Addiction Scale—Short Version SAS-SV

The Smartphone Addiction Scale (SAS) is a validated screening tool measuring excessive smartphone use among adolescents and young adults (Kwon et al., 2013; for Italian validation, see De Pasquale et al., 2017). The short version of SAS is composed of 10 questions rated on a dimensional scale from 1 “completely disagree” to 6 “strongly agree.” The total score ranges from 10 to 60, and higher scores correspond to a higher level of smartphone use.

### Statistics Analysis

Four sets of statistical analysis were conducted. First, to investigate group differences in the ability to produce completed and elaborated solutions on social problems, a set of independent *t*-tests were performed on all subscales of Roberts-2 test. Given that Problem Identification Scales (PID) and Resolution Scales (RES) are mutually exclusive subscales, a composite score for each of them was computed. Following Parolin et al. (2019), the composite score was calculated by multiplying the odds obtained in each scale with their order and adding the resulting scores (i.e., RES1 + RES2 * 2 + RES3 × 3 + RES4 × 4 + RES × 5). Second, correlation analysis was computed to investigate the relationship between the ability to process social information and resolve social problems and the perception of thriving and well-being at school. Third, to investigate differences in problematic smartphone use, an independent *t*-test was run on the scores obtained from the SAS-SV questionnaire for the two groups. Finally, correlation analysis was planned to further explore the relationship between social cognitive functioning (Roberts-2) and problematic smartphone use.

Precisely due to the relatively small sample, Spearman correlations were computed between the scores obtained in the Roberts-2 and the scores obtained by CIT, the Student Engagement Scale, and SAS-SV for each group.

## Results

### Social Cognitive Functioning

**FIGURE 1:**
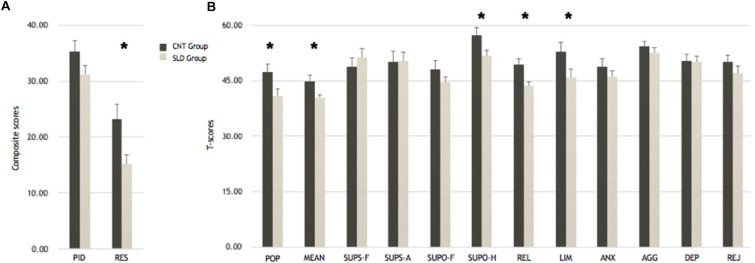
Results from *t*-tests comparison on Roberts test scores between groups. **(A)** Composite scores calculated on raw scores obtained in PID and RES scales. **(B)** Comparison on T-scores obtained in cognitive psychological functions and emotional scales. ^∗^*p* ≤ 0.05.

Differences between SLD and the control group were assessed by means of an independent *t*-test on the T-scores in each scale of Roberts-2 (Parolin et al., 2019). Specifically, the *t*-test comparison revealed that the control group obtained higher T-scores than the SLD group in POP (CNT group: *M* = 47.37, *SD* = 9.51; SLD Group: *M* = 40.89, *SD* = 8.18; *t*(36) = 2.25, *p* = 0.031, *d*’ = 0.75) and in MEAN (CNT group: *M* = 45.00, *SD* = 6.52; SLD group: *M* = 40.37, *SD* = 4.34; *t*(36) = 2.58, *p* = 0.014, *d*’ = 0.86). Furthermore, the CNT group showed a greater ability to resolve a social problem positively and with an increased level of elaboration (RES Composite) than SLD group (CNT: *M* = 23.21, *SD* = 12.22; SLD: *M* = 15.21, *SD* = 7.52; *t*(36) = 2.43, *p* = 0.020, *d*’ = 0.81). Moreover, the *t*-tests revealed a reliable difference between groups in the scales related to external support as SUPO-H and REL. Specifically, the control group score was higher than SLD group score in both SUPO-H (CNT: *M* = 57.42, *SD* = 9.85; SLD: *M* = 51.79, *SD* = 6.75; *t*(36) = 2.06, *p* = 0.047, *d*’ = 0.69); and REL (CNT: *M* = 49.47, *SD* = 7.38; SLD: *M* = 43.74, *SD* = 5.08; *t*(36) = 2.79, *p* = 0.008, *d*’ = 0.93). Finally, the control group obtained a significantly greater score in LIM than SLD (CNT: *M* = 52.95, *SD* = 11.05; SLD: *M* = 45.95, *SD* = 10.57; *t*(36) = 2.00, *p* = 0.054, *d*’ = 0.67). No other effect was found (*p*_*s*_ > 0.132). Participant scores for each scale are shown in Figure 1.

### Social Cognitive Functioning and Well-Being

In order to investigate whether a different functioning in social competence was related to well-being, Spearman correlations analysis was conducted between the Roberts-2 scales (POP, MEAN, RES-Composite, SUPO-H, REL, and LIM), the scores obtained in CIT 8 subscales, and the scores of the 4 subscales of Student Engagement Scale separately for each group. Concerning holistic well-being, a significant positive correlation was found in the CNT group between SUPO-H and Engagement-CIT [*r*_*s*_(19) = 0.470, *p* = 0.042] and also with Total-CIT [*r*_*s*_(19) = 0.467, *p* = 0.044]. Similarly, a significant positive correlation was found in the SLD group between REL and Meaning-CIT, [*r*_*s*_(18) = 0.505, *p* = 0.032]. No other significant effects were found in the CNT group (*p*_*s*_ > 0.130) and in the SLD group (*p*_*s*_ > 0.080). Concerning school engagement, the analysis for the CNT group revealed that RES-Composite and SUPO-H correlated positively with Affective Engagement [*r*_*s*_(17) = 0.534, *p* = 0.027; *r*_*s*_(17) = 0.628, *p* = 0.007]. On the contrary, the scores obtained in LIM by the SLD group correlated negatively with the Cognitive Engagement Scale [*r*_*s*_(18) = −0.561, *p* = 0.015]. No other significant effects were found in the CNT group (*p*_*s*_ > 0.095) and in the SLD group (*p*_*s*_ > 0.084).

#### Social Cognitive Functioning and Smartphone Use

Differences between groups in problematic use of smartphones was measured by an independent *t*-test. No significant differences were found between the CNT group (*M* = 28.28, *SD* = 10.42) and the SLD group (*M* = 29.58, *SD* = 8.66), *t*(35) = −0.414, *p* > 0.005.

To further explore the relationship between different psychosocial functioning profiles raised by Roberts-2 and smartphone use, Spearman correlations between the scores obtained in POP, MEAN, RES-Composite, SUPO-H, REL, and LIM Roberts-2 scales and self-report SAS-SV were run for each group. Concerning the CNT group, the analysis revealed that POP, MEAN, RES-Composite, and REL were negatively linked to smartphone abuse [*r*_*s*_(18) = −0.455, *p* = 0.058; *r*_*s*_(18) = −0.474, *p* = 0.047; *r*_*s*_(18) = −0.541, *p* = 0.020; *r*_*s*_(18) = −0.572, *p* = 0.013]. No other significant correlations were found either for the CNT group (*p*_*s*_ > 0.206) or for the SLD group (*p*_*s*_ > 0.324).

## Discussion

Although several studies have shown the presence of socio-cognitive and emotional difficulties in SLD children, these aspects have been less investigated in adolescence. Moreover, relatively little research has analyzed the connections between social functioning, well-being, and smartphone use through both performance-based and self-report measures. Previous studies (Bauminger et al., 2005; Bauminger-Zviely et al., 2019) have demonstrated the presence of difficulties in SLD students in understanding and solving social situations, abilities that support a more general social competence. Our findings partially overlap with previous research and deepen the knowledge about this area of functioning. Specifically, our results show significant differences between adolescents with and without a diagnosis of SLD in terms of the functions that underlie the ability to tell a complex and articulated story, such as recognize, explain, and resolve a problematic social situation. Specifically, the SLD group was less able to identify the problem depicted in the picture, to provide adaptive and complex solutions to it, and to produce complete stories by selecting and organizing the main elements in the pictures. These results indicate that SLD students have difficulties in perceiving everyday social situations accurately and adaptively and in understanding the process by which social problems can be solved. These abilities are essential to behave appropriately in a social context, to understand other people’s behavior, and, therefore, to succeed in creating supportive relationships. As far as social support is concerned, our data suggest that SLD students perceive the social environment as less helpful and trustworthy. As stated before, this can be partially explained by parent, teacher and classmate perceptions of SLD students that is frequently negative and can worsen the self-esteem and self-efficacy of these students, making it even more difficult to ask and receive help (Livingston et al., 2018). However, our results don’t allow us to establish the nature of these social difficulties, which should be the topic of further studies. Understanding the processes underlying social functioning can be highly informative, especially in SLD students. Indeed, the perception of support by others and the feeling that it is possible to ask for help when needed are aspects that tap into established relational expectations and are key resources when facing problems in life. This is especially true in school, which is both a learning and a social context, where the possibility to ask and receive help is fundamental to overcome day-to-day challenges. Research has revealed that these abilities affect SLD achievement more than for their peers, especially in higher grades of education (Trainin and Swanson, 2005). In addition to this, the significant difference on the Limit Setting Scale between groups suggests that SLD students perceive the adult world as less able to administer fitting consequences for problem behaviors. A recent study on parental styles suggested that parents of SLD children experience higher levels of distress (Bonifacci et al., 2016) and struggle to maintain a stable parental style, possibly moving from granting independence to over-reactivity. These results are particularly meaningful if compared to the fatigue reported by teachers in dealing with students with special needs, and the students’ perceptions of a worse relationship with their teachers (Tobia and Marzocchi, 2015). The difficulties felt by the adult world in handling children with SLD can partially explain our results, as they could be responsible for the perception of a less constructive presence of parents and teachers.

Contrary to the literature (i.e., Mugnaini et al., 2009), adolescents with SLD did not show a higher level of internalizing problems compared to typically developing peers. However, it should be noted that the literature is not consistent in reporting evidence for these problems in the SLD population (e.g., Miller et al., 2005), suggesting that the difference between SLD and control groups might be sensitive to the assessment battery and to environmental factors, such as receiving an intervention and an early diagnosis (e.g., Mugnaini et al., 2009). Moreover, the lack of internalizing problems may, at least in part, be explained by the age of the sample. Adolescence is an emotionally challenging period for every student, so it is less likely that a difference would be found due to increased individual variability in both groups. Unfortunately, our data do not enable us to specify what type of mechanism may underpin the processing of emotion in the SLD population, and further investigations are needed.

Concerning the second hypothesis, the results suggest a similar profile of general well-being functioning for both SLD and control students. An increasing sense of thriving is related to growing trust and perceived support from others. In particular, the possibility to ask for help when needed and to trust others seems more important for SLD adolescents, while a key for the control group is the role played by the concrete help provided by the social environment. On the contrary, school engagement seems to be related to different aspects of socio-cognitive functioning in SLD and control groups. Existing literature has pinpointed a link between parent, peer, and teacher support and school affective engagement (Furrer and Skinner, 2003; Estell and Perdue, 2013), emphasizing the role of support from others as a fundamental school commitment predictor. This is precisely what we found in typically developing adolescents. Our study further enhances these results, as it identifies positive relations between affective school engagement and a deep understanding of the process underlying the resolution of a social situation. This is of fundamental importance, as the class is a social context as well as an academic one and learning how to interact successfully with peers and adults is an essential developmental task that is deeply connected with school engagement. This relation seems absent in SLD students, for whom other aspects are more relevant to school engagement. Notably, their investment in learning processes decreases the more they perceive parents and teachers as ready to impose rules and limits for their shortcomings. This is interesting given that SLD students perceive external regulation less than their peers, however, when they do, it seems complicated to attribute a constructive meaning to it. Unfortunately, our results do not allow us to determine the direction of this result.

Turning to our third hypothesis, our results do not highlight a significative difference between groups in terms of smartphone use. Nevertheless, the correlations between socio-emotional functioning and problematic smartphone use highlight a different functioning in the two groups. In typically developing adolescents, increasing smartphone use seems related to a decrease in perceived support by others, which is confirmed by recent studies on this topic (Wang et al., 2017; Herrero et al., 2019). Furthermore, smartphone use is negatively related to social-cognitive variables, such as the ability to produce an increasingly complex solution to social problems taking into consideration all the main elements. This result links problematic smartphone use not only with social support but also with more in-depth aspects of social understanding and interpretation. Surprisingly, this pattern was not found in SLD students, whose scarce perception of social support seems unrelated to the use of the smartphone. To the best of our knowledge, researchers have not yet addressed this issue, so we can only argue that the diminished social support and trust in others may be a problem for SLD that does not depend on problematic smartphone use, but that is probably due to other variables, such as parenting styles (Bonifacci et al., 2016) and/or self and social-stigma associated with SLD (Chan et al., 2017). To note, the main limitation of this study is the relatively small sample size; therefore, our results are to be interpreted carefully and confirmed by further investigations.

While the present study takes into consideration just some of the variables involved in socio-cognitive and emotional functioning, it contributes to our understanding of psychosocial variables relevant to adolescents with SLD. It also highlights the relevance of a better understanding of socio-emotional functioning, especially in adolescents with SLD, in order to improve their academic experience and, in general, their quality of life.

## Data Availability Statement

The datasets generated for this study are available on request to the corresponding author.

## Ethics Statement

The studies involving human participants were reviewed and approved by the Ethics Committee of Catholic University of the Sacred Heart. Written informed consent to participate in this study was provided by the participants’ legal guardian/next of kin.

## Author Contributions

DS has conceived the study, has predisposed the clinical test to be administered and the inclusion criteria for enrollment of the subjects, as supervisor of the study and discussed the results. RB was responsible for collecting the data, data analysis, drafting of all sections of the manuscripts and critical revision of the final version of the manuscript in collaboration with IO. MT has contributed to the data collection and coding (narrative test). EL has contributed to the data collection and coding (self-report). DT has contributed to the definition of self-report. ML led the discussion on test and results.

## Conflict of Interest

The authors declare that the research was conducted in the absence of any commercial or financial relationships that could be construed as a potential conflict of interest.
